# A sham case-control study of effectiveness of DTP-Hib-hepatitis B vaccine against rotavirus acute gastroenteritis in Kenya

**DOI:** 10.1186/1471-2334-14-77

**Published:** 2014-02-11

**Authors:** Sammy Khagayi, Jacqueline E Tate, Reuben Onkoba, Umesh Parashar, Frank Odhiambo, Deron Burton, Kayla Laserson, Daniel R Feikin

**Affiliations:** 1 Kenya Medical Research Institute (KEMRI)/Centers for Disease Control and Prevention (CDC) Research and Public Health Collaboration, Kisumu, Kenya; 2 National Center for Immunizations and Respiratory Diseases, Centers for Disease Control and Prevention, Atlanta, GA, USA; 3 International Emerging Infections Program, Global Disease Detection Division, Center for Global Health, Centers for Disease Control and Prevention, Nairobi, Kenya; 4 Center for Global Health, Centers for Disease Control and Prevention, Atlanta, GA, USA; 5 Division of Preparedness and Emerging Infections, Centers for Disease Control and Prevention, Atlanta, GA, USA

**Keywords:** Rotavirus, Case-control, Vaccine effectiveness

## Abstract

**Background:**

In many GAVI-eligible countries, effectiveness of new vaccines will be evaluated by case-control methodology. To inform the design and assess selection bias of a future case-control study of rotavirus vaccine effectiveness (VE) in western Kenya, we performed a sham case-control study evaluating VE of pentavalent vaccine (DTP-Hib-HepB) against rotavirus acute gastroenteritis (AGE).

**Methods:**

From ongoing rotavirus surveillance, we defined cases as children 12 weeks to 23 months old with EIA-confirmed rotavirus AGE. We enrolled one community-based and two hospital-based control groups. We collected vaccination status from cards at enrollment, or later in homes, and evaluated VE by logistic regression.

**Results:**

We enrolled 91 cases (64 inpatient, 27 outpatient), 252 non-rotavirus AGE facility-based controls (unmatched), 203 non-AGE facility-based controls (age-matched) and 271 community controls (age-matched). Documented receipt of 3 pentavalent doses was 77% among cases and ranged from 81-86% among controls. One percent of cases and 0-2% of controls had no pentavalent doses. The adjusted odds ratio of three versus zero doses for being a case was 3.27 (95% CI 0.01-1010) for community controls and 0.69 (95% CI 0.06-7.75) for non-rotavirus hospital-based AGE controls, translating to VE of -227% and 31%, respectively, with wide confidence intervals. (No facility-based non-AGE controls were unvaccinated.) Similar results were found for ≥2 pentavalent doses and for severe rotavirus AGE.

**Conclusions:**

The study showed that it is feasible to carry out a real case control in the study area, but this needs to be done as soon as the vaccine is introduced to capture the real impact. Sham case-control or pilot studies before vaccine introduction can be useful in designing case-control VE studies.

## Background

Rotavirus causes approximately 453,000 deaths annually in children, most of them occurring in sub-Saharan Africa and Asia [1]. By December 2012, 13 of 73 countries eligible for funding through the Global Alliances for Vaccines and Immunizations (GAVI) had introduced rotavirus vaccines into their national immunization programs and 20 more countries, including Kenya, had been approved to receive GAVI funding for rotavirus vaccine [2,3].

As more developing countries introduce rotavirus vaccines, there is growing interest in evaluating their impact to support decisions for continued and augmented country-level financing. In many countries with on-going rotavirus surveillance, rotavirus vaccine effectiveness (VE) will be evaluated by case-control methodology [4-6]. Case-control studies for evaluation of VE have the advantage of being relatively quick, not requiring pre-vaccine data or a well-defined denominator population, and being efficient in the setting of rare diseases [6,7]. Case-control studies have been utilized to evaluate rotavirus VE in Nicaragua and El Salvador [8-10], as well as other vaccines in various settings [7,11-13]. While having advantages, case-control studies are inherently at risk of selection bias due to the challenges of selecting a sample of controls representative of the population from which the cases arose [7,14,15]. If controls differ in their propensity to be vaccinated or to seek medical care from the source population of cases, then the association between vaccine and disease can be distorted, leading to biased estimates of VE [7,16]. Besides selection bias, case-control studies can suffer from misclassification of exposure (i.e. vaccination status).

We used ongoing surveillance for rotavirus gastroenteritis in rural western Kenya to perform a case-control study evaluating effectiveness of pentavalent vaccine (diphtheria, pertussis, tetanus *Haemophilus influe*nzae type b (Hib), and hepatitis B) on rotavirus gastroenteritis. Since pentavalent vaccine should have no protection against rotavirus disease, we refer to this study as a “sham” case-control study, and as such, after adjusting for potential confounders, any “vaccine effectiveness” could be ascribed to residual selection bias, and could be useful in interpreting results of a real case-control study of rotavirus VE undertaken after the vaccine’s introduction into Kenya. Besides assessing selection bias, our study aimed to define characteristics of the population (e.g. vaccine coverage and timeliness) and the design (e.g. ability to document vaccine status) that would inform the design and execution of a real case-control study of rotavirus vaccine after its introduction into Kenya.

## Methods

### Rotavirus surveillance

The study was conducted in Siaya County, western Kenya, in the setting of a long-standing Health and Demographic Surveillance System (HDSS) [17]. The area is rural, poor, has high malaria and HIV prevalence, and a high under-5 mortality ratio [17-19].

Surveillance for acute gastroenteritis (AGE) among children <5 years of age was conducted among inpatients at a district hospital and outpatients at two health centers located in the HDSS area. AGE was defined as ≥3 looser than normal stools and/or >1 episode of unexplained vomiting followed by loose stool within a 24-hour period beginning within seven days before seeking healthcare. Stool was collected in a plastic diaper from which at least 2 ml of stool was scooped into a specimen container. In the outpatient facilities, if the child was unable to provide stool during the visit, a field worker would follow-up at the child’s home to collect stool. All stool samples were collected within 48 hours after presentation, stored and transported in cool boxes on the same day of collection to KEMRI/CDC laboratories, approximately 60 kilometers away. Batch testing for rotavirus using enzyme-immunoassay (EIA, Rotaclone™ Kit) was done.

### Case-control study

The case-control study was embedded in ongoing rotavirus surveillance system. Eligible children were 12 weeks (i.e. 2 weeks after the second scheduled Expanded Program for Immunization visit) to 23 months of age who presented with AGE, as these children are eligible to have received pentavalent vaccine. Among these, cases were defined as those with an EIA-confirmed rotavirus-positive stool sample. Severe AGE was defined using the 20-point numerical Vesikari scoring system with scores of ≥11 [20]. Case selection at the sites was consecutive until the desired sample size was reached. Inpatients were enrolled continuously, including during nights and weekends. Outpatients were enrolled during weekday daytime hours.

We enrolled two hospital and one community-based control groups. The first hospital control group was unmatched, consisting of children presenting to the same facility as the cases, who had AGE not caused by rotavirus, as determined by a negative rotavirus EIA test. The second hospital control group was children presenting with a non-AGE illness, excluding those who had other EPI-vaccine-preventable diseases (i.e. measles, pneumonia, meningitis, sepsis, otitis media, bacteraemia, epiglottitis, hepatitis and pertussis). In a 3:1 ratio, controls were age-matched to cases within -15 to +60 days of the case-patient’s birthday. Eligible controls were enrolled starting from the admission closest in date to the case’s admission date, until three controls were enrolled. The third control group was selected from the same villages as the case-patients, but not living in the same compound. Starting at the case’s compound, we went to the nearest compound on either side of the case’s compound to check for eligible children. If no children met eligibility criteria in that compound, we went to the adjacent compound in the same direction until we enrolled a control. After the first control, we skipped the next compound and inquired about eligible controls at the following compound. We repeated this process until we enrolled three controls.

### HIV testing

HIV testing was done using parallel rapid test kits on whole blood obtained from finger-prick --Determine HIV-1/2 (Abbott Diagnostic Division, Hoofdorp, Netherlands) and Bioline® (Standard Diagnostics Inc, Korea) - with tie-breaking of discordant results using Unigold™ (Trinitiy Biotech PLC, Ireland). PCR testing confirmed HIV status for children <18 months old who were antibody-positive on at least one rapid test.

### Data collection

During an interview with trained study staff, the parent was asked for each child’s Ministry of Health-issued vaccination card. For children who came to the hospital without their vaccination card, we visited their home and collected the immunization history from available cards. If the card was not available, a verbal report of the child’s vaccination status was collected. Other data collected included history, symptoms and signs of illness for cases, parent’s education level, household characteristics and possessions, and distance to the nearest health facility.

### Sample size

For the sham case-control study, we did not expect to find statistically significant “effectiveness” of the pentavalent vaccine against rotavirus AGE, but rather to determine if the study found a point estimate that gave an indication of potential bias (i.e. the odds ratio was significantly different from 1.0). To estimate enrollment duration for a real case-control study, we determined the sample size based on a rotavirus VE evaluation. While coverage with two Pentavalent doses is 94% in the area [21], we assumed that in the first year of rotavirus vaccine introduction, coverage among infants would be lower due to obstacles of rolling out the vaccine. We therefore estimated 50% coverage. Using these parameters, we needed 61 cases and183 controls in each of the three control groups (3:1 control to case ratio) to detect a VE of 60% in the first year of life. To account for missing data, we increased the sample size by 10%, to 67 cases and 201 controls per control group.

### Data analysis

A dose of pentavalent was considered valid (i.e. immunologically protective) if it was administered ≥14 days before the date of admission for the case, 14 days before the date of admission for the case for the matched controls and 14 days before the control child’s admission for the unmatched hospital controls.

To evaluate socioeconomic status (SES), we constructed quintiles based on eight indicator variables of asset ownership which were scored and reduced to a single score through principal component analysis [22]. We considered risk factors for rotavirus AGE using community controls only because comparison between cases and the two hospital control groups evaluated rotavirus AGE risk against other diagnoses, which has less meaningful epidemiologic interpretation. Comparison between rotavirus AGE cases and hospital controls was done only to identify variables for adjustment in logistic regression.

The primary analysis included only documented reports of vaccine status; having zero doses was determined from either vaccination card or verbal report as it was expected that unvaccinated children may be less likely to have cards, which are usually given at the first vaccination in the clinic. The main analysis compared children who had received three doses versus zero doses. Secondary analyses included children who had received at least two documented doses versus zero doses. For the analysis of the matched control groups, conditional logistic regression was used; all variables with a p value of <0.10 in univariate analysis were incorporated in multivariable analysis using backwards elimination. Variables with more than 5% unknown responses were included in the model with “unknown” coded as a category of a dummy variable. Children with unknown vaccine status were always excluded from the model. VE was calculated as ((1-Odds Ratio) X 100). We also did an unmatched analysis of cases and non-rotavirus facility-based AGE controls, controlling for age in months and seasonality (4 quarters of the year) using unconditional logistic regression. We reran the analysis for only severe cases. Lastly, we evaluated timely vaccination, defined as having received the vaccine between one week before and two weeks after the scheduled date of vaccination, which in Kenya is 6, 10 and 14 weeks for the primary EPI series.

### Ethical considerations

This study was approved by the Kenya Medical Research Institute’s ethics review committee (SSC#1836). Written informed consents were obtained from all participants or their legal guardians prior to enrolment.

## Results

**Figure 1:**
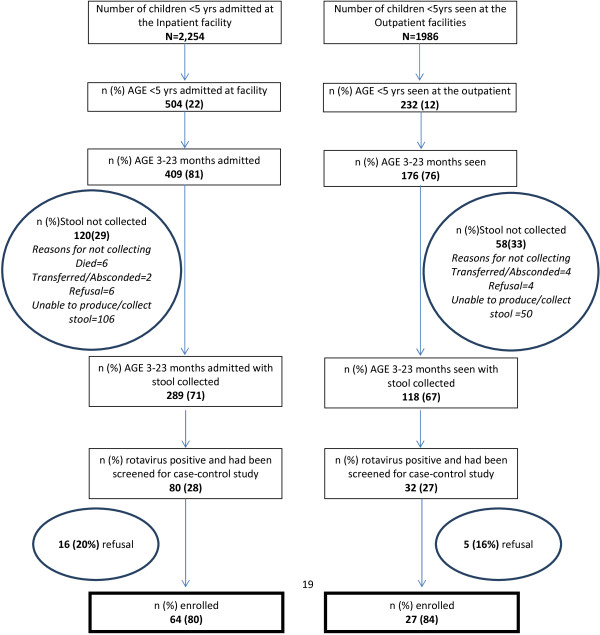
Flow chart of rotavirus surveillance and enrollment of cases in case–control study from December 2010 to November 2011.

Case enrollment occurred from December 1, 2010 through November 30, 2011. There were 507 AGE hospitalizations among children <5 years old and 232 AGE sick visits in the outpatient facilities, of which, 409 (81%) and 176 (76%) were among children 3–23 months of age, respectively (Figure 1). We obtained stool specimens from 289 (71%) age-eligible inpatients and 118 (67%) age-eligible outpatients. Severe AGE occurred in 197 (68%) inpatients and 35 (30%) outpatients (Table 1). Among inpatients, stool samples were more likely to be collected from children <12 months of age and with severe AGE (Table 1). No difference in stool collection was seen among outpatients. Among the 3–23 month old inpatients and outpatients with AGE who had stool collected, 80 (28%) and 32 (27%), respectively, were positive for rotavirus (Figure 1). Five (6%) hospitalized rotavirus-positive cases died during their admission.

**Table 1:** Characteristics of children with AGE with and without stool collection, western Kenya, 2010-11

	**Inpatients with AGE (n = 409)**			**Outpatients with AGE (n = 176)**		
	**Stool collected, n (%)**	**Stool not collected, n (%)**	**P value**	**Stool collected, n (%)**	**Stool not collected, n (%)**	**P value**
Age						
3-11 months	196 (68)	67 (56)	0.02	78 (66)	31 (53)	0.10
12–23 months	93 (32)	53 (44)		40 (34)	27 (47)	
Sex						
Male	160 (55)	59 (49)	0.25	71 (60)	29 (50)	0.20
Female	129 (45)	61 (51)		47 (40)	29 (50)	
Vesikari score						
≥11	197 (68)	68 (57)	0.03	35 (30)	18 (31)	0.85
<11	92 (32)	52 (43)		83 (70)	40 (69)	
HIV status						
Positive	18 (6)	9 (8)	0.17	2 (2)	0 (0)	0.58
Negative	227 (79)	84 (70)		53 (45)	28 (48)	
Unknown	44 (15)	27 (22)		63 (53)	30 (52)	
HDSS resident						
Yes	183 (63)	68 (57)	0.21	116 (98)	55 (95)	0.15
No	106 (37)	52 (43)		1 (1)	3 (5)	

Among age-eligible rotavirus AGE cases, 64 (80%) inpatients and 27 (84%) outpatients were enrolled (Figure 1). We enrolled 252 AGE hospital controls without rotavirus detected, 203 non-AGE hospital controls and 271 community controls. In multivariable analysis, comparing rotavirus AGE cases to community controls having a father with less education , a mother currently married, and being moderately-to-severely stunted were protective against being a case, while being in the least poor SES quintiles and not attending daycare were significant risk factors (Table 2).

**Table 2:** Univariate analysis comparing risk factors among rotavirus AGE cases and controls, western Kenya, 2010-11

**Variable**^**a**^	**Cases (N = 91)**	**Community controls (N = 271)**			**Hospital non-AGE controls (N = 203)**			**Hospital non-rotavirus AGE controls (N = 252)**		
	**n (%)**	**n (%)**	**Univariate P value**	**Multivariate OR (95% CI)**	**n (%)**	**Univariate P values**	**Multivariate OR (95% CI)**	**n (%)**	**Univariate P values**	**Multivariate OR (95% CI)**
Male	53 (58)	136 (50)	0.26		102 (50)	0.34		135 (54)	0.44	
Age in months^b^										
3–5	31 (34)	67 (25)		Matched	47 (23)		Matched	60 (24)	ref	
6–11	36 (40)	120 (44)			92 (45)			96 (38)	0.28	
12–17	16 (18)	47 (17)			47 (23)			58 (23)	0.08	
19-23	8 (9)	37 (14)			17 (8)			38 (15)	0.05	
Mother ≤ primary education	66 (74)	222 (82)	0.10		161 (80)	0.16		204 (82)	0.14	
Father ≤ primary education^c^	39 (43)	174 (64)	<0.01	0.32 (0.16 – 0.68)	102 (50)	0.02	0.30 (0.13 – 0.70)	147 (58)	<0.01	0.44 (0.25 – 0.80)
Mother currently married	68 (76)	229 (85)	0.08	0.15 (0.04 – 0.66)	167 (83)	0.08	0.30 (0.10 – 0.87)	210 (83)	0.15	0.31 (0.14 – 0.66)
≤ 30 minutes to health facility^c^	48 (53)	127 (47)	0.53		102 (50)	0.17		115 (46)	0.29	
SES quintile										
Poorest	17 (20)	80 (30)	<0.001	1	41 (21)	ref	1	45 (18)	ref	
Poorer	6 (7)	58 (22)		0.75 (0.24 – 2.34)	34 (17)	0.15	0.17 (0.04 – 0.76)	53 (21)	0.02	
Poor	12 (14)	56 (21)		0.96 (0.36 – 2.56)	32 (16)	0.62	0.54 (0.17 – 1.78)	47 (19)	0.36	
Less poor	34 (40)	48 (18)		2.54 (1.11 – 5.85)	54 (27)	0.38	1.27 (0.46 – 3.50)	65 (27)	0.36	
Least poor	17 (20)	25 (9)		2.00 (0.75 – 5.35)	38 (19)	0.91	0.56 (0.16 – 1.97)	34 (11)	0.50	
HIV positive	3 (3)	5 (2)	0.01		7 (3)	ref		15 (6)	Ref	
HIV negative	64 (70)	231 (85)			160 (79)	0.54		164 (65)	0.30	
HIV unknown	24 (26)	35 (13)			36 (18)	0.45		73 (29)	0.46	
Born prematurely^c^	2 (2)	11 (2)	0.60		25 (12)	0.03	0.13 (0.02 – 0.83)	36 (14)	0.01	
Source of water										
Tap	9 (10)	13 (5)	0.03		17 (8)	ref		7 (3)	ref	1
Unprotected spring	33 (32)	80 (30)			76 (37)	0.43		84 (33)	0.03	0.23 (0.07 – 0.75)
River/stream	38 (42)	118 (44)			73 (36)	0.34		114 (45)	0.01	0.21 (0.07 – 0.66)
Other sources	11 (12)	58 (22)			37 (18)	0.16		47 (19)	0.01	0.13 (0.03 – 0.47)
Ever used ORS before	70 (80)	183 (69)	0.03		133 (67)	0.03	3.12 (1.27 – 7.64)	203 (82)	0.88	
Does not attend daycare	84 (92)	219 (82)	0.01	4.14 (1.41 – 12.15)	192 (95)	0.83		224 (89)	0.36	
Season										
Jan-Mar	54 (59)	164 (61)		Matched	130 (64)		Matched	95 (38)	ref	1
Apr-Jun	20 (22)	55 (20)			42 (21)			101 (40)	<0.01	0.27 (0.14 – 0.52)
Jul-Sep	10 (11)	31 (11)			12 (6)			29 (12)	0.22	0.63 (0.27 – 1.46)
Oct-Dec	7 (7)	20 (7)			19 (9)			27 (11)	0.09	0.39 (0.15 – 1.05)
Underweight										
Normal	77 (84)	212 (78)	0.15		154 (76)	ref	1	181 (72)	ref	
Moderate	10 (11)	37 (14)			18 (9)	0.72	0.57 (0.17 – 1.93)	29 (12)	0.59	
Severe	4 (4)	22 (8)			31 (15)	0.01	0.10 (0.02 – 0.60)	42 (17)	0.01	
Stunting										
Normal	64 (70)	119 (44)	<0.01	1	128 (63)	ref		160 (63)	ref	
Moderate	12 (13)	54 (20)		0.60 (0.25 – 1.46)	33 (16)	0.35		41 (16)	0.39	
Severe	15 (16)	98 (36)		0.19 (0.08 – 0.48)	42 (21)	0.05		51 (20)	0.35	
Wasting										
Normal	84 (92)	252 (93)	0.91		171 (84)	ref		194 (77)	ref	
Moderate	4 (4)	8 (3)			14 (7)	0.34		21 (8)	0.14	
Severe	3 (3)	11 (4)			18 (9)	0.12		37 (14)	0.01	

^a^Only variables that were significantly different between cases and controls, or of particular epidemiologic interest, are listed in tables. The following variables were not significantly associated with case status: maternal age, number of children in household, fare to nearest facility, history of an AGE hospitalization, enrollment in the HDSS, treats drinking water, and mother knows about ORS.

^b^Two controls that were < 3 months old and three that were >23 months old, due to range of allowable matching ages were included in the 3–5 and 19–23 age groups respectively.

^c^> 5% persons with missing or unknown responses included in multivariable analysis as a dummy variable (see methods).

The percentage of cases with documented vaccination status was 83%, which was lower than among community controls (90%) and higher than hospital controls (74-77%). Among cases, 77% had received 3 doses of pentavalent by documented report and only 1% were unvaccinated (Table 3). Among control groups, this proportion with 3 doses varied slightly -- 86% for community controls, 81% for facility-based non-AGE controls, and 83% for unmatched facility-based AGE controls, according to the vaccine card with only 0% - 2% unvaccinated.

**Table 3:** Table comparing immunization status among rotavirus AGE cases and controls, western Kenya, 2010–11

**Variable**^**a**^	**Cases (N = 91)**	**Community controls (N = 271)**	**Hospital Non-AGE controls (N = 203)**	**Hospital AGE controls unmatched (N = 252)**
	**n (%)**	**n (%)**	**n (%)**	**n (%)**
Number of pentavalent doses received^b^				
0 doses	1 (1)	3 (1)	0 (0)	3 (2)
1 dose	4 (5)	5 (2)	7 (5)	7 (4)
2 doses	12 (16)	25 (10)	20 (14)	23 (12)
3 doses	58 (77)	210 (86)	118 (81)	160 (83)
Of those with 2 doses of pentavalent^c^				
Dose 2 received on time	47 (67)	152 (65)	89 (64)	121 (66)
Dose 2 not received on time	23 (33)	83 (35)	49 (36)	61 (34)
Of those with 3 doses of pentavalent^c^				
Dose 3 received on time	30 (52)	117 (56)	61 (52)	90 (56)
Dose 3 not received on time	28 (48)	93 (44)	57 (48)	70 (44)

^a^First response to each variable was referent category in conditional logistic regression.

^b^A dose was considered given if it was given at least 14 days before the date of admission of the cases for cases and matched controls while for the unmatched controls it was 14 days before their date of admission. Timely vaccination considered vaccination <1 before or >2 weeks after scheduled date of pentavalent2 pentavalent3 at 10 weeks and 14 weeks of age respectively.

^c^Unknown/missing responses not included.

The adjusted odds ratio of three doses versus zero doses was 3.27 (95% CI 0.01-1010) for community controls and 0.69 (95% CI 0.06-7.75) for facility-based AGE controls; this translates to VE of -227% and 31%, respectively, with very wide confidence intervals (Table 4). (No facility-based non-AGE controls were unvaccinated, thus this analysis could not be performed.) Similar results were found when evaluating ≥2 doses of pentavalent vaccine versus zero doses and when restricting cases to severe rotavirus AGE (Table 4).

**Table 4:** Univariate and multivariable analysis comparing immunization status for rotavirus AGE cases versus controls, western Kenya, 2010–11 (documented immunization status only)

**Variables**^**a**^	**Community controls OR (95% CI)**		**Hospital non-AGE controls OR 95% CI**		**Hospital AGE controls**^**c **^**(unmatched) OR 95% CI**	
	**Unadjusted OR**	**Adjusted OR**^**a**^	**Unadjusted OR**	**Adjusted OR**^**b**^	**Unadjusted OR**	**Adjusted OR**^**c**^
3 versus 0 pentavalent doses	1.00 (0.08 – 11.93)	3.27 (0.01 – 1010)	Undefined	Undefined	1.09 (0.11 – 10.66)	0.69 (0.06 – 7.75)
≥2 versus 0 pentavalent doses	0.77 (0.07 – 8.55)	2.26 (0.06 – 865)	Undefined	Undefined	1.15 (0.12 – 11.22)	0.64 (0.06 – 6.79)

Missing and unknown responses for variables were excluded in the regression.

^a^Regression with community controls adjusted for father’s education, mother’s marital status, socioeconomic status of household, child attends daycare and stunting.

^b^Regression with hospital non-AGE controls adjusted for father’s education, mother’s marital status, socioeconomic status of household, child born prematurely, child ever treated with ORS and underweight.

^c^Regression with hospital AGE controls adjusted for father’s education, mother’s marital status, season of the year and source of water.

**Figure 2:**
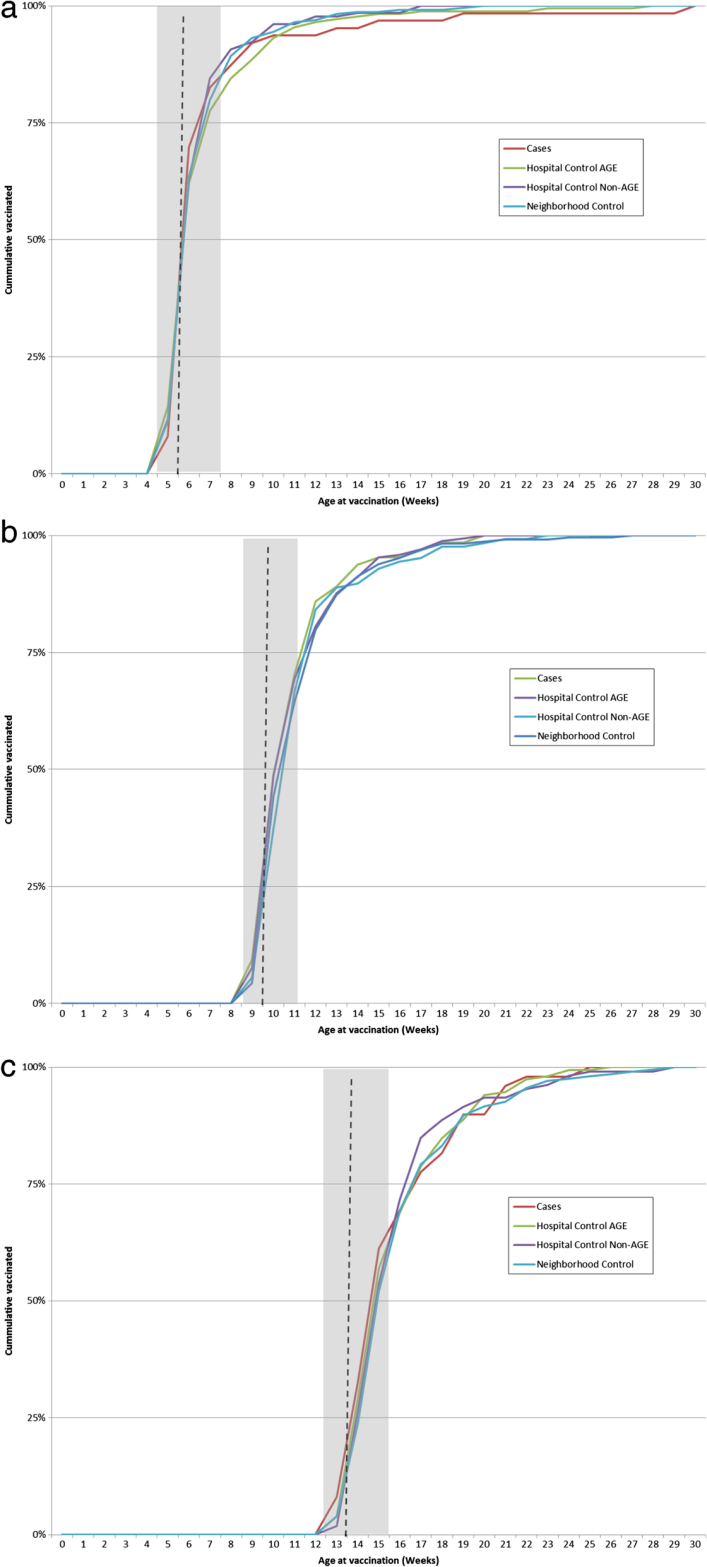
**Distribution of age of vaccination for dose 1, 2 & 3 of pentavalent vaccine by cases and controls. a, b, c** Vertical dotted line represents scheduled date at 6, 10 & 14 weeks. Grey shaded area is considered timely vaccination.

Among cases with documented timing of pentavalent immunization, 83%, 70% and 69% received timely vaccination for pentavalent doses 1, 2 and 3, respectively (Figure 2). Similar timing of vaccination was seen among all control groups.

## Discussion

Our sham case-control study provided several important lessons that can be applied to a case-control study of rotavirus VE in the same setting after vaccine introduction, expected in 2014 [2]. First and foremost, a major challenge was finding very few unvaccinated children, which limited the study’s power to make any conclusions about bias. Given the high coverage levels of routine immunizations in Kenya, unvaccinated children may not be epidemiologically representative of children in this population. An evaluation of rotavirus VE in Nicaragua found that small differences in vaccine coverage resulted in substantially disparate estimates of VE when coverage was high (≥95% vaccinated), but not when coverage was lower (~91% vaccinated) [23]. To avoid this problem in Kenya, we plan to perform the case-control study of rotavirus vaccine during the roll-out phase before full coverage is achieved.

Although case-control studies often employ multiple control groups in an attempt to understand bias, multiple control groups often yield varying effectiveness estimates, leaving researchers with challenges in interpretation of the results. We explored three different source populations for enrolling controls. Children in all three control groups were largely similar to case-patients with regard to potentially confounding socio-demographic characteristics. One exception was that the hospitalized control groups tended to have more severe malnutrition, suggesting children hospitalized with non-rotavirus illnesses tend to be different in their nutritional status, which can potentially confound VE estimates.

The ease of enrollment differed between the three control groups. Matched non-AGE facility-based controls were easy to enroll, but difficult to find sufficient numbers to meet matching criteria and harder to collect vaccination data. Community control enrollment achieved sufficient numbers, but required additional expense and logistical coordination. Children hospitalized for AGE who tested negative for rotavirus offered a time and resource-efficient alternative to the other two more traditional control groups, because they were already being tested through the diarrheal surveillance platform. These children had the same healthcare seeking behavior for diarrheal illness as individuals with rotavirus diarrhea, and had the most similar demographic profile to rotavirus AGE cases, particularly SES which can be related to health-seeking and vaccination. Additionally, study staff were blinded to the child’s case or control status at time of enrollment and during vaccine verification, which occurred before EIA results were available. The rotavirus EIA is highly sensitive and specific and thereby minimizes the misclassification of cases and controls [24-26]. Based on these reasons, we believe in this setting the use of test-negative controls will provide the most resource efficient and epidemiologically unbiased control group, and should be the only control group for the real case-control study.

Another challenge was documenting vaccination status and dates of vaccination. The proportion of children for whom we were unable to document vaccination status ranged from 10% in community controls to 29% in the non-AGE hospital controls. High levels of undocumented vaccination status could bias VE estimates particularly if vaccination status is differentially missing by number of doses received. For the real case-control study, efforts will be made to seek documentation of vaccination for all cases and controls in the home or the immunization clinics, when unavailable in the hospital.

We did not find any significant associations of pentavalent vaccination on rotavirus gastroenteritis in children. There are several possible explanations. First, a lack of association in the absence of selection bias is expected as pentavalent vaccine should confer no immunologic protection against rotavirus disease. Therefore, it is possible that there was no selection bias. Second, as mentioned, our study had very limited power due to the low numbers of both case and control children who received no pentavalent doses. Confidence intervals around VE estimates were very wide and included 1 for all evaluable control groups. Sham case-control studies, like real ones of vaccine effectiveness, are not useful in settings of extremely high vaccine coverage. Third, our study might have been biased because many children were excluded from the analysis because they did not have their vaccination cards available and/or had unknown vaccination status and this percentage varied between cases and controls. If these children systematically tended to be more or less vaccinated, we might have missed an association between pentavalent vaccine and rotavirus AGE.

A similar approach to quantifying bias in case-control studies is the so-called “bias-indicator” study in which the cases are children hospitalized with non-rotavirus AGE, a syndrome for which rotavirus vaccine should have no protective efficacy so that any association between the vaccine and case-status is likely due to selection bias [6,27]. Bias-indicator studies are a different type of sham case-control study than we performed, in that the sham is not the exposure (i.e. vaccine) but rather the outcome (i.e. type of gastroenteritis). This approach was taken in evaluations of effectiveness of oral cholera vaccine in Mozambique and rotavirus vaccine in the U.S., both indicating no measurable selection bias [27,28]. The advantage of the bias-indicator approach is that it can be done contemporaneously with an actual case-control study of rotavirus AGE cases, which cannot be done for a sham case-control study like ours due to the co-administration of most EPI vaccines.

We found almost a third of children received the second and third pentavalent doses late. Late vaccination could have implications for rotavirus vaccine, as young infants who are unvaccinated are at the greatest risk of severe dehydration from rotavirus, particularly early in introduction of vaccine before herd protection occurs [23]. In addition, concerns persist about increased absolute risk of intussusception among children given late rotavirus vaccination, although WHO acknowledges that the risk of severe rotavirus disease outweighs any potential increased risk of intussusception among late vaccines [29-31]. Emphasis not just on vaccination, but on timely vaccination, is important in developing countries, where median ages of vaccination tend to be weeks to months behind schedule [32]. Moreover, for the case-control study, late vaccination could bias towards increased likelihood of vaccination among controls if the age-matching window is too wide. In this study, we allowed children to be matched who were up to 60 days older than the case, which is probably too wide; we will decrease that window for age-matching in the real case-control study.

## Conclusions

Monitoring the impact and performance of new vaccines, like rotavirus vaccine, in routine immunization programs is important for countries and can be used to advocate further support for the immunization program. Case-control studies remain one of the more accessible methods for such evaluations, despite their limitations. The findings from this sham case-control study will enable future rotavirus vaccine case-control studies in Kenya to be designed more efficiently. Sham case-control studies of VE or pilot studies of existing vaccination patterns in communities should be considered prior to the start of other case-control studies of the target vaccine to assess the existence of selection bias and improve the study design and efficiency.

## Abbreviations

AGE: Acute gastroenteritis; EIA: Enzyme immunoassay; GAVI: Global alliance for vaccines and immunization; HDSS: Health and demographic surveillance system; HIV: Human immunodeficiency virus; OR: Odds ratio; PCR: Polymerase chain reaction; VE: Vaccine effectiveness.

## Competing interests

The authors declare that they have no competing interests.

## Authors’ contributions

SK, JT, UP, FO, KL and DF designed the study. SK, RO and FO collected and managed the data. SK, JT, KL and DF analyzed data and drafted the manuscript. DB and UP revised the manuscript. All authors read and approved the final manuscript.

## Pre-publication history

The pre-publication history for this paper can be accessed here:

http://www.biomedcentral.com/1471-2334/14/77/prepub
